# Psychological Research on Sleep Problems and Adjustment of Working Hours during Teleworking in the COVID-19 Pandemic: An Exploratory Study

**DOI:** 10.3390/ijerph192114305

**Published:** 2022-11-02

**Authors:** Sandra Figueiredo, Raquel João, Laura Alho, João Hipólito

**Affiliations:** 1 Department of Psychology, Psychology Research Centre (CIP), Universidade Autónoma de Lisboa, Foundation for Science and Technology (FCT), 1169-023 Lisbon, Portugal; 2 Department of Psychology, Universidade Autónoma de Lisboa, 1169-023 Lisbon, Portugal; 3 Think Wise, 3810-133 Aveiro, Portugal; 4 Mind—Clinical and Forensic Institute, 1990-019 Lisbon, Portugal; 5 Department of Psychology, Psychology Research Centre (CIP), Universidade Autónoma de Lisboa, 1169-023 Lisbon, Portugal

**Keywords:** health, exposure to screens, teleworking, COVID-19, sleep quality, gender differences, psychology

## Abstract

Mandatory home isolation caused by COVID-19 in professional contexts led to a situation that required work activities to be converted into a remote modality. The literature on this topic is very recent, given the pandemic and the uncertainty of virtual and face-to-face work modalities. This study aimed to examine the effects of adults’ prolonged exposure to screens on sleep quality, the type of devices used according to age and gender, periods of access to such devices and the impact on performance in the context of telework due to COVID-19. Specifically, the study analyzed the differences in the use of devices and in the time spent using them during and after teleworking between genders and age groups. A total of 127 Portuguese participants answered the Pittsburgh Sleep Quality Index and a questionnaire that we specifically developed to characterize teleworking habits. The results showed differences between men and women regarding the use of devices and its impact on sleep quality, as well as differences in terms of age. These results are discussed in terms of how the current work context may affect performance, sleep, gender differences and the adverse effects of exposure to screens during and after work hours.

## 1. Introduction

The pandemic that was officially declared in March 2020 throughout the world due to the coronavirus has radically changed population habits. Mandatory home isolation and conditional freedom in many professional contexts led to a situation that required work activities to be converted into a remote modality. In 2022, we are still facing restrictions regarding home isolation and telework (ICT-based technologies) restrictions in several countries, with various studies elucidating the impact of telework on people’s well-being and family dynamics [1,2,3]. The modus vivendi also changed in Portugal. Measures to contain the virus focused on teleworking (home isolation) to avoid contagion. Currently, despite the slowdown of the pandemic and the implementation of vaccination, telework, in many cases, continues to prevail in organizations and family circles. The return to the face-to-face modality has been poorly managed (by workers) with regard to sleep habits and daytime performance levels due to the long period of telework they are used to [4,5,6,7,8].

Telework, even when integrated in a dual system of face-to-face and remote modalities, has had implications that were analyzed by scientific research from 2020–2022 in terms of mental and physical health [9,10,11]. These measures are representative of the main forms of work and new social rules around the world as a result of several waves of COVID-19. In this work, we examined family contexts involving a new model of living in which several members are adapted to home confinement, with online work and school activities in the same space and at the same time. Sleep has been addressed considering the impact of telework and age/gender differences [5,7,12,13], but the evidence is still poor.

Considering the family context and work at home, the literature suggests that women and men behaved differently in terms of telework before the pandemic outbreak [12]. Women face less autonomy with remote work due to family constraints and the more roles they play in family tasks (working at home full-time). In terms of sleep, telework and gender, there is no consensus yet. However, the tendency is that females suffer more from sleep instability due to telework and COVID-19 restraints [5,13]. In addition, there are conflicting results observed among the current studies. On the one hand, some have shown that sleep was not affected by telework during the COVID-19 pandemic, and on the other hand, other studies proved a statistically significant relationship between poor sleep and telework during the pandemic [5,7,13,14]. Among the latest studies, research on sleep and telework (also involving gender, age and location variables) is scarce, and more evidence is needed to understand how sleep, cognition and telework are really functioning. The different populations in these studies and their respective geographies (Brazil, Switzerland, USA and Portugal) should be noted. Furthermore, previous studies identified gender segmentation in the telework context. The time and types of tasks are different among males and females, with advantages (longer sleep duration, better quality of sleep, and more time for other tasks that are not work-related) for males. However, organizations and their cultures are the main factor in understanding how autonomy is managed by both genders engaged in telework. Families with children have more difficulties managing telework, with the main focus on women [11,15,16,17].

After the COVID-19 outbreak, studies continued to find the same evidence confirming that women experience more difficulties compared to men [18]. The main complaint is due to the increased working hours with telework. Lizana et al. [19] reported that a sample of teachers declared that their work periods increased during the pandemic in the telework context with adverse effects on the quality of life in the family context. Likewise, the authors reported differences based on gender, with mostly negative effects for females (in their work–family–mental-health balance). A similar situation was observed in Lithuanian [20] and Portuguese samples [13].

Another complaint is the reduced time control due to family supervision at home (concerning child care) and also the increased use of smartphones during work. The timetables for work and “non-work” periods became difficult to differentiate. The schedules changed within families, with more effort for females with children at home (schooling online simultaneously). Considering the remote modality, women and men with children at home (up to 16 years old) reported less productivity at work and decreased quality of life [21]. In contrast, women without children and in the telework setting showed high levels of productivity and quality of life [21].

From another perspective, scholars from Malaysia revealed findings about the positive perceptions of employees (males and females) toward telework or the online work environment [22]. On the contrary, work-to-life and life-to-work were perceived as a stressor by Italian employees. Working in the office was preferred to the home-based situation [23]. For example, women tended to prefer open spaces and suffered more from the “home-office” situation compared to men [24]. This last study was conducted before the pandemic struck. It is not surprising that these gender-related perceptions and the abrupt shift from the office to teleworking from home are directly related to the quality-of-life-changes that have occurred since the pandemic situation [7,20]. Advantages for quality of life were addressed differently in specific geographies during and before this pandemic [24].

The use of smartphones and their impact on reducing time control have already been referred to. Considering gender differences, there is no significant work that has explored how women and men differ in their use of electronic devices during telework. The basic principle is that women face disadvantages using technology in work settings compared with men [25,26,27]. However, this type of data does not contribute to understanding how females and males are affected by devices used during telework, mainly considering that women are often overloaded at home with family and domestic tasks [28]. Even considering this assumption—that women perform telework simultaneously with family care in the household—investigations into telework in the COVID-19 context are scarce.

However, on the other hand, it is important to understand the impact of electronic devices and telework itself on mental and physical health [29,30,31,32]. Psychological problems are more often addressed in the literature by comparing them to physical problems. Depression, anxiety and work overload are the most common symptoms identified by individuals [26,27]. Sleep problems were found in recent studies considering telework and the increased use of smartphones during the lockdown and post-home-isolation period, but with scarce evidence [26,28]. On the other hand, recent studies revealed the advantages of telework when correlated with satisfactory indices of mental health (and work productivity, despite busier schedules), especially for females [32,33,34]. Other studies revealed no significant associations between working from home and mental health issues [35].

This study aimed to investigate the behavior of adults regarding the use of electronic devices during and after working hours during home isolation in 2020/2021, with expected implications for changes in sleep schedules and work performance. These implications were analyzed according to age and gender differences.

## 2. Materials and Methods

### 2.1. Hypotheses

This study was designed to analyze the specificities of the effect of increased exposure to screens on sleep quality, more or less prolonged periods of using electronic devices during and after work (especially before bedtime) and performance. This analysis treated gender and age as independent variables. Thus, the following hypotheses were tested: (1) men and women differ in the use of devices during telework, which determines different periods of exposure to screens between genders; (2) men and women have different levels of sleep quality and require different amounts of “time to fall asleep”; (3) organization and work performance are negatively related to the increased use of screens in the context of home isolation (telework); and (4) the method of use of electronic devices and the time spent using electronics before bedtime vary based on age.

### 2.2. Participants

Only subjects over 18 years old participated in the study. They were in the telework system and are Portuguese, living in Portugal. There was no compensation for participating in the study. A total of 127 participants were validated for the study sample after checking the instruments: 74 females (58.3%) and 53 males (41.7%). The mean age was 37.99 (SD = 9.54; the minimum age was 21 years, and the maximum age was 68 years). For marital status, 49 participants were single (38.6%), 61 were married (48.0%), 9 were divorced (7.1%) and 8 were in a de facto union (6.3%). Regarding education, 4 of the participants had primary education (3.1%), 21 had secondary education (16.5%), 4 had completed a higher professional technical course (3.1%), 54 had a bachelor’s degree with honors (42.5%), 31 had a master’s Degree (24.4%), 5 had a doctorate (3.9%), 6 had a postgraduate certificate (4.7%) and 2 had an ordinary bachelor’s degree (1.6%). With regard to teleworking, 50 were teleworking on a part-time basis (teleworking/on-site) (39.4%), and 77 were teleworking full-time (60.6%).

### 2.3. Instruments

#### 2.3.1. Sociodemographic Questionnaire and “Screen Exposure”

A questionnaire entitled “Screen exposure” was designed to assess the increased exposure to screens variable, including open questions such as: “What type of digital media devices do you use in telework?”; “How long, on average, are you exposed to these devices when teleworking?”; and dichotomic questions such as “Do you feel that due to increased exposure to screens you have more difficulty sleeping?”. These questions were entered as variables in the correlation test in order to identify the subjects’ behavior towards each item.

For the devices used in telework (by day), 37 of the participants used a computer (29.1%), 3 used a cell phone (2.4%), 67 used a computer and cell phone (52.8%), only 1 used a cell phone and tablet (0.8%), 15 used a computer, cell phone and tablet (11.8%), 2 used a computer, cell phone and television (1.6%) and 2 used a computer, cell phone, television and tablet (1.6%). As for the time spent in front of screens during telework, 15 of the participants were exposed for periods of 3 to 5 h (11.8%), 43 were exposed for 5 to 8 h (33.9%), 60 were exposed for 8 to 12 h (47.2%) and 9 were exposed for more than 12 h (7.1%). Regarding exposure to screens (over a 24-hour period), 57 participants had more difficulty sleeping (44.9%), and 70 of the participants said they did not experience increased difficulty sleeping (55.1%). Regarding the use of devices after work, 126 answered that they used them (99.2%), and only 1 did not use devices after work (0.8%).

With regard to devices used after work, 26 participants used a cell phone (20.5%), 3 used television (2.4%), 14 used a computer and cell phone (11%), 1 used a computer and television (0.8%), 5 used a cell phone and tablet (3.9%), 43 used a cell phone and television (33.9%), 1 used a tablet and television (0.8%), 1 used a computer, cell phone and tablet (0.8%), 16 used a computer, cell phone and television (12.6%), 7 used a cell phone, tablet and television (5.5%) and 9 used a computer, cell phone, tablet and television (7.1%).

As for the question about the use of electronic devices just before bedtime, 98 said yes (77.2%) and 29 said no (22.8%). Of the participants who said yes, 41 responded that they had more difficulty sleeping (32.3%), and 57 responded that they did not have increased difficulty sleeping after using electronic devices (44.9%).

#### 2.3.2. Pittsburgh Sleep Quality Index

The Pittsburgh Sleep Quality Index was developed by Buysse et al. [36] and was validated for the Portuguese population by João et al. [37]. This instrument was created with the aim of providing a reliable, valid and standardized measure of sleep quality by differentiating two types of sleepers (good and poor). It was also developed to provide an easy-to-complete questionnaire for patients and to enable reliable interpretation by mental health professionals and researchers alike. It consists of 19 items divided into 7 components: (1) subjective sleep quality, (2) sleep latency, (3) sleep duration, (4) usual sleep efficiency, (5) sleep disorders, (6) use of medication to sleep (7) and dysfunction during the day. These components are rated on a scale of 0 to 3 [33]. Regarding the internal consistency of the instrument, the original version presented α.83 (Cronbach’s alpha), and the Portuguese version was α.70 [36]. In this study, not all of the instrument’s questions were used because some questions were intended only to obtain clinical information, which was not the main goal of the study [36]. Therefore, this study only presents scores for sleep quality, which is classified as “very good” (1), “good” (2), “poor” (3) and “very poor” (4) (Q2), and the time—in minutes and referring to the last thirty days—it takes the person to fall asleep after going to bed (Q6).

#### 2.3.3. Procedures

For the administration of the instruments and research conduct, the authors adhered to the Committee on Publication Ethics (COPE) standards. After approval of the research project by the Ethics Committee of Universidade Autónoma de Lisboa, data collection began in 2021. The participants were given different instruments (see Instruments section). Their prior informed consent with the guarantee of confidentiality and anonymity was obtained, and they were informed about the objective of the study and the instruments necessary to carry out the research. The questionnaires were completed online or with paper/pencil. The entire procedure was in accordance with the ethical principles of the Declaration of Helsinki and in accordance with the European Union’s ethical standards.

#### 2.3.4. Data Analysis

For the statistical analysis of the data, the Statistical Package for Social Sciences (SPSS) version 26.0 (IBM Corp, Armonk, NY, USA) for Windows was used. First, the homogeneity of the sample was tested considering all variables under analysis, and the Levene test confirmed the homogeneity of the variance with regard to the dependent variables vis-à-vis the groups under analysis (female and male). Therefore, population variances were similar in both gender groups (*p* >0.05). To assess the normality of the sample distribution, the Kolmogorov–Smirnov test was conducted. Therefore, parametric tests were carried out to examine the effects of the dependent variables of this research. Then, we ran repeated univariate analyzes to compare groups (determined by the independent variables gender and age) with regard to the frequency of the use of electronic devices during working hours and after working hours, specifically around bedtime, in the context of home isolation. The effect size (Cohen’s d) values were addressed.

## 3. Results

### 3.1. Comparison between Groups Involved in Teleworking during the Pandemic: Gender and Prolonged Exposure to Screens

Univariate analyses were performed to compare gender and age groups with regard to the frequency of the use of electronic devices during working hours and after working hours, specifically around bedtime, in the context of home isolation. Tukey values from the multivariate analyses revealed an overall significant group effect. Differences in this dependent variable between the groups were prominent (F (7.716) = 123.943; *p* = 0.006), which means that women used more devices after teleworking (M= 7.22; Standard Error = 3.886), and men had prolonged exposure to screens during the telework period (here, the exposure extent refers to the number of hours during the work period, not after telework, as occurred in women) (M = 0.23; Standard Error = 0.176). Therefore, H0 was rejected for the first hypothesis of the study. On the other hand, statistically significant differences were not seen in other variables related to the use of electronic devices during working hours (at home). Regarding the use of devices after work per gender group, the effect sizes (Cohen’s d) of the differences were medium to high (0.59).

No significant differences were detected in work performance/organization between gender groups (see Table 1).

From the descriptive results of the group statistics, women had greater difficulty sleeping because of their exposure to screens during the teleworking period. They used more electronic devices after teleworking compared to males. Women also handled screens more after the working period.

### 3.2. Correlation between Variables during Telework in the Pandemic: Age, Exposure to Screens and Sleep

The adults who reported “greater difficulty in sleeping” (caused by prolonged exposure to screens during the day and outside work) were statistically significantly influenced by greater “use of devices before bed”. Older participants reported greater use of devices while teleworking compared to younger participants (*p* < 0.05).

As for the type of devices: older people used a greater number of devices, such as a computer and cell phone at the same time, at work. In terms of age, the older they were, the more they used devices before bed and after work (*p* < 0.0001; r −0.31). On the other hand, the younger they were, the greater the difficulty they felt in falling asleep after using devices in the context of the pandemic and telework (*p* < 0.0001; r −0.33). Age and performance had a positive and significant correlation (*p* < 0.05; r −0.18).

The correlation coefficients (Pearson) revealed that the quality of sleep (good or poor sleep) was negatively and significantly correlated (*p* < 0.05; r −0.21) with difficulty before sleeping in the context of telework/screens/isolation, which was expected. That is, the worst sleep quality reported was associated with greater difficulty before sleeping. With regard to the time it takes for the subject to fall asleep (Q2), the correlations were similar: a negative and significant association between difficulty sleeping and using electronic devices (*p* < 0.05). In addition, as expected, the relationship was positive and significant—*p* < 0.05; r −0.18)—between sleep quality (a high score indicates a worse sleep level on a scale of 1 to 4 of the Pittsburgh Index) and exposure/prolonged use of screens during teleworking (see Table 2 and Table 3).

In relation to telework, the performance and organization of work were also analyzed, and it is interesting to examine the relationship between these variables and the use of devices during telework. The results showed a positive and significant relationship between screen use and performance (*p* < 0.05; r −0.18), as well as between performance and work organization (*p* < 0.05; r −0.66), as reported by the subjects. This relationship between performance and task organization was expected. However, the use of electronic devices positively enters into this relationship, although with less of an effect (r −0.18 and r −0.66).

## 4. Discussion

Given the first hypothesis of the study, it was confirmed that adult men and women differ in the use of devices in the context of the pandemic and teleworking conditions, which explains the different periods of exposure to screens between genders. The differences were statistically significant in two periods regarding the use of screens: during and after teleworking. Women were the most exposed to screens and exposed on a prolonged basis after the work period. Women’s prolonged use of devices “out of hours” with direct implications for sleep is a relationship that follows the same trend described in studies by Nash et al. and Rozman et al. [38,39] and those of Lizana, Raisiene and Crepaldi [7,19,20]. In addition, females in the present study used more devices before going to bed. On the other hand, men were revealed to have more exposure (in the time frame considering the screens used) during the work schedule (referring to the number of hours facing a screen during telework compared to clerical work with less screen and ICT usage).

In general, the aforementioned studies found that women showed higher levels of stress, lower income and professional dissatisfaction, and worse sleep behaviors (especially regarding flexible periods compared to sleep patterns before teleworking and the pandemic) when compared to men in the remote work context [5,6,7,40,41].

These data, which are much different between genders and characterized by new work methodologies during the pandemic, are also corroborated by previous studies [19,24,42,43] and by an OECD Report [44], which show that work habits are different between men and women because of an additional cause: women tend to report more work at home due to parental care and the supervision of children, including in the context of home isolation (remote schooling). Thus, the present study shows a drawback for women in that they are more devoted to domestic tasks, but they also use more devices and work more outside of the telework period, with adverse effects on their sleep habits. On the contrary, other recent studies [12] proved that women with no children benefit (better performance and fewer sleep complaints) from telework or ICT-based work.

Excessive work with electronic devices without a time limit has another implication, which was the focus of the second hypothesis of our study: negative effects on sleep times, specifically the delay in sleeping periods related to the difficulty that the Portuguese women in our study experience after hours of teleworking and using devices beyond official working hours. These data are supported by previous studies [45,46] because women (not Portuguese: one of the studies [45] was conducted in Poland, and the majority of the sample—more than 80%—was female) experienced high indices of daily fatigue (caused by sleep disruption and depression symptoms) during the pandemic. Insomnia was not significant for the women assessed [45]. In Sinha’s study [46], gender and age were assessed as independent variables. The results showed that sleep was affected—especially the onset and waking periods—during the lockdown, and there was increased use of electronic devices in males. In addition, as observed in our study, males used more electronic devices (during the telework schedule). However, in our study, the females used more digital devices before going to sleep, with an impact on their sleep onset.

This relationship—long hours teleworking + screens with prolonged exposure and after hours + impaired sleep quality + difficulty falling asleep + female gender—was also confirmed by other recent studies [47,48].

With regard to the third hypothesis, we cannot confirm it because teleworking was not negatively related to performance or organization, as perceived by the subjects. In this context, there were no significant differences between gender and work performance/organization (but rather between age and performance), as confirmed by the chosen statistical tests for comparison. However, other results indicated a positive relationship between performance and the use of electronic devices during telework, given the COVID-19 contingency. Performance, according to the correlation test, appears to be significantly associated with the organization of work, which is coherent and expected. These data conflict with the studies by Moretti et al. [47], because job satisfaction was reported as having a negative relationship with performance. These indices have been mainly linked to aspects of mental health impairment, which has been frequently mentioned by workers teleworking during the COVID-19 pandemic [49]. This context is still very prominent in Portugal and in other countries for reasons of virus containment. However, our results do not agree with the literature published in 2020 and 2021 on the relationship between telework and dissatisfaction/disorganization at work.

Other studies [50] revealed that working remotely allowed for higher job satisfaction rates, as workers could choose their own working conditions and comply with their schedules. These conditions include workspace, regulated temperature, the possibility of sharing the house with teenage children and better experience (quality of life) with the family due to the possibility of better managing the schedule from home. This perspective has not been replicated in the pandemic context, which is probably due to the stress and uncertainty generated by the pandemic. In the case of situations where workers’ homes do not allow silence and regulated periods for work/family/sleep, the situation worsens and has implications on physical and mental health, as reported in the study by Moretti et al. [47] and in various studies conducted since 2020 on this topic [8,27,28,33,34].

Our results with this sample in Portugal are in good agreement with the study by Samani [42]. To compare another study in the context of the pandemic, we can mention, for example, Rieth and Hagemann [47]. The data from this German study point to the advantages that workers see in working from home, in contrast to disadvantages and work disorganization during COVID-19 in a face-to-face work context. It is likely that the performance, satisfaction and organization perceived by the subjects depend on the cultural factor and age [48] and also on the variable COVID-19 scenario in each country.

Kooji [48] and Vyas and Butakhieo [49] presented meta-analyses and interpretive perspectives on self-regulation at work in home confinement during COVID-19, posing two main questions that deserve further examination: age and telework; telework and its permanent possibility as a solution. However, there is a lack of consensus among the results of studies on this topic because researchers have had only two years to carry out research. However, the abrupt adaptation to telework was felt differently, mainly by the older adult population. Older workers faced greater challenges, but this did not necessarily mean underperformance or problems in engaging with the job. On the contrary, in our study, we found that younger subjects reported more satisfactory performance during prolonged periods of teleworking during the pandemic, but with implications regarding sleep habits.

According to Vyas and Butakhieo [49], Hong Kong is considering reasonable for making telework permanent, given the positive solutions obtained despite the pandemic. For the optimal performance and regulation of well-being, authors tested the effect of mindfulness techniques on populations in the initial period of the lockdown [50], indicating that such techniques helped to maintain the well-being of people at home in an active professional context.

We totally reject, therefore, the third hypothesis of our study because work organization and performance were not negatively related to the prolonged use of screens while in home confinement. This assumption, based on the coefficients, is supported by other previously identified studies that showed evidence regarding the correlation between the use of screens (implicit telework variable) during telework and high satisfaction/performance. This satisfactory correlation can also be explained by the efforts of the intervention last year to promote the well-being of populations teleworking and in home confinement during COVID-19 [51].

Finally, the fourth hypothesis, “subjects will differ according to age in the use of electronic devices during telework and in the frequency of use before going to bed”, was confirmed. Younger-aged people in the sample denoted greater problems in falling asleep due to prolonged exposure to screens of devices used during the day and before bedtime. On the contrary, the older they were, the more they used devices compared to younger subjects. In addition, poorer sleep quality was associated with greater difficulty before sleeping. This diversity and differences in the use of technologies between age groups have already been seen to have a consequence on sleep quality and job satisfaction when comparing children and adults, usually family members of the observed children [52,53]. More time spent exposed to screens was negatively related to sleep quality [5,6,7]. Younger people with prolonged access to smartphones after school assignments showed greater problems in sleep quality [54,55]. Similar results were found in our study.

Younger subjects complained the most about falling asleep in relation to prolonged exposure to electronic devices. From another perspective on the use of devices, older participants reported a greater number of devices than younger ones, such as computers, tablets and cell phones [56]. Potentially, the handling of fewer devices (telework using a specific selection of electronic devices) and a shorter exposure time to screens may have influenced the positive result for performance, as self-assessed by the younger subjects. This is in terms of performance and considering the correlation coefficients for age x performance. These data are in accordance with data from previous research [55], which pointed to sleep problems and changes in rhythms and schedules during successive lockdown periods [56], focusing on the age variable. Specifically, it was also confirmed that younger subjects faced greater sleep problems (falling asleep and waking up) compared to older subjects [57,58]. In the European Union Report of Lodovici [59], younger workers have struggled more with telework since the beginning of the COVID-19 outbreak.

Longer periods of sleep were experienced by older adults in Spain [60], with positive results during the lockdown. Younger adults usually have younger children, and this factor may explain the greater difficulty falling asleep in the younger adult population in home isolation. This assumption is supported by the results of the study by Kahn et al. [60]. Additionally, the younger population was also identified as the age group (especially those up to 35 years old) whose sleep schedules were most affected due to home isolation and telework.

In general, all data obtained in this study, including the correlation of poor sleep quality with difficulty before sleeping, were expected and are in line with recent studies [8,10,16]. A relationship between telework, long hours in front of screens and disturbed sleep, especially considering the chronotype, affecting evening-type persons more than morning ones, was identified in populations studied by Merikanto et al. [61]. On the other hand, the impact of telework and age on sleep is due to exposure to screens during after-work hours, that is, unplanned hours, which became common in the pandemic [62,63]. Therefore, the fact that late hours are harmful to proper functioning at work and to the emotional balance of populations has been evidenced in the recent literature [63,64].

The result for the correlation between poor sleep quality and prolonged exposure/use of screens during telework was expected considering the corpus of data obtained. Longer sleep periods have actually been identified and associated with teleworking and prolonged exposure to screens during the day, as recent studies attest [65].

Due to COVID-19, the exposure time to screens has been increasing, as well as the proximity of screens to eyes, for many people. This may be due to the need for people to learn and work at home. Employees and students spend more time in front of screens that emit levels of blue light that can be harmful. Thanks to the increased amount of time spent on digital devices in the COVID-19 period, eye-care professionals and employers are increasingly concerned about the impacts they may have on the health of the population due to increased exposure to blue light [66]. According to several authors, increased time exposed to screens impacts optical health, diet, habits and sleep routines [67,68,69].

According to Afonso, Fonseca and Teodoro [33], full-time teleworkers have high levels of anxiety and depression, particularly very high scores with regard to poor sleep quality compared to other studies carried out throughout the pandemic. Exposure to natural light during the day and to artificial light at night, as well as exposure to artificial light and screens, has an impact on individuals’ sleep thanks to their effect on the production of melatonin and the regulation of circadian rhythms. Exposure to light at night is associated with depressive symptoms, while using devices with screens is associated with insomnia [33].

In short, most of our hypotheses were confirmed, with the exception of the third one, as work organization/performance and the use of screens (verified impact) were not negatively related. The relationship between sleep (specifically sleep quality) and age deserves further analysis given the data obtained and the difference between younger and older persons. This analysis should focus on the current remote or mixed online/in-person work context, the geography of the subjects and the pandemic scenario.

## 5. Conclusions

Through a repeated series of univariate analyses and calculated correlation coefficients, the results make an empirical contribution to understanding how the current work context of populations is affecting performance, work organization, sleep and periods of exposure to screens during and after work while in home isolation due to the COVID-19 pandemic.

It is also important to examine how this exposure to screens and its implications for sleep in groups, determined by age and gender, will behave in the current period of transition between online and in-person work modalities, with the mixed mode prevailing. We conclude that men and women differ in the use of devices during telework, with women using fewer devices but having more difficulty falling asleep. In terms of age, younger adults use fewer devices and have better performance but experience less positive sleep quality (especially with regard to falling asleep) compared to older ones. On the contrary, the income and organization of work were not affected by telework. As for study limitations, the sample could be larger, but only the part of the sample that completely responded to all instruments was considered. In future studies, it will be important to cover more regions of the country, as well as more countries, for a comparative and cross-cultural study. As in any exploratory study, we point out some limitations that can be considered in further studies. First, we did not use any objective measures to assess the exact effects of telework on sleep, which is important for a more comprehensive view of such effects.

Furthermore, we did not assess the existence of previous clinical conditions, such as depression or anxiety, which are known to have an impact on sleep quality. A symptom inventory and structure scales and questionnaires would be appropriate to investigate the effects of such conditions, adding more relevant information and strengthening our results in several ways.

Lastly, regarding variables of interest that should be further examined in future studies, the geographic factor, the educational level and the labor system are some examples [9,28,29]. Additionally, the quality of life related to telework should be evaluated concerning quality time with children, child leisure and the quality of life with partners at home.

## Figures and Tables

**Table 1:** Gender behaviors regarding exposure to screens during and after teleworking.

Origin	Dependent Variable	Type III Sum of Squares	gl	Mean Square	F	Partial Eta Squared	Observed Power
Corrected model	Work organization	5.738	1	5.738	0.482	0.004	0.106
	Performance	0.920	1	0.920	0.064	0.001	0.057
	Fulfillment Power	2.112	1	2.112	0.134	0.001	0.065
	Involvement	9.252	1	9.252	0.603	0.005	0.120
	C7somatory	0.340	1	0.340	0.207	0.002	0.074
	C5somatory	26.316	1	26.316	1.098	0.009	0.180
	Devices during telework	32.245	1	32.245	2.803	0.022	0.383
	Devices after work	123.943	1	123.943	7.716	0.059	0.787
Intercept	Work organization	88,961.167	1	88,961.167	7467.681	0.984	1.000
	Performance	89,372.857	1	89,372.857	6230.974	0.980	1.000
	Fulfillment Power	87,391.827	1	87,391.827	5545.243	0.978	1.000
	Involvement	84,707.506	1	84,707.506	5521.125	0.978	1.000
	C7somatory	431.610	1	431.610	262.217	0.679	1.000
	C5somatory	6666.316	1	6666.316	278.038	0.692	1.000
	Devices during telework	2875.388	1	2875.388	249.927	0.668	1.000
	Devices after work	8261.086	1	8261.086	514.303	0.806	1.000
Gender	Work organization	5.738	1	5.738	0.482	0.004	0.106
	Performance	0.920	1	0.920	0.064	0.001	0.057
	Fulfillment Power	2.112	1	2.112	0.134	0.001	0.065
	Involvement	9.252	1	9.252	0.603	0.005	0.120
	Devices during telework	32.245	1	32.245	2.803	0.022	0.383

**Table 2:** Age, use of devices, sleep quality and work performance.

		Devices during Telework	Time Spent with Devices during Telework	Difficulty Sleeping Caused by Telework	Difficulty Sleeping Caused by Devices Used before Sleep	Devices Used before Sleep
Type of devices used during telework	Pearson	1	0.091	0.152	0.153	0.093
	Sig. 2-way		0.311	0.088	0.132	0.297
	N	127	127	127	98	127
Time using devices during telework	Pearson	0.091	1	−0.134	−0.132	−0.151
	Sig. 2-way	0.311		0.132	0.196	0.090
	N	127	127	127	98	127
Increased difficulty sleeping	Pearson	0.152	−0.134	1	0.570 **	0.076
	Sig. 2-way	0.088	0.132		0.000	0.396
	N	127	127	127	98	127
Increased difficulty sleeping	Pearson	0.153	−0.132	0.570 **	1	
	Sig. 2-way	0.132	0.196	0.000		0.000
	N	98	98	98	98	98
Devices used before sleep	Pearson	0.093	−0.151	0.076		1
	Sig. 2-way	0.297	0.090	0.396	0.000	
	N	127	127	127	98	127
Age	Pearson	0.207 *	−0.153	0.066	0.052	0.306 **
	Sig. 2-way	0.020	0.086	0.462	0.614	0.000
	N	127	127	127	98	127
Sleep quality	Pearson	0.032	0.180 *	−0.331 **	−0.209 *	−0.007
	Sig. 2-way	0.719	0.042	0.000	0.039	0.940
	N	127	127	127	98	127
Time delay before sleep	Pearson	−0.146	−0.028	−0.484 **	−0.392 **	−0.148
	Sig. 2-way	0.102	0.755	0.000	0.000	0.096
	N	127	127	127	98	127
Work organization	Pearson	0.206 *	0.019	−0.045	−0.151	−0.168
	Sig. 2-way	0.020	0.833	0.618	0.137	0.058
	N	127	127	127	98	127
Performance	Pearson	0.180 *	−0.091	−0.054	−0.117	0.007
	Sig. 2-way	0.043	0.306	0.544	0.251	0.938
	N	127	127	127	98	127

* Correlation is significant at the 0.05 level (2-way); ** Correlation is significant at the 0.01 level (2-way);

**Table 3:** Age, use of devices, sleep quality and work performance.

	Age		Sleep Quality	Time Delay before Sleep	Work Organization	Performance
Type of devices used during telework	Pearson	0.207 *	0.032	−0.146	0.206 *	0.180 *
	Sig. 2-way	0.20	0.719	0.102	0.020	0.043
	N	127	127	127	98	127
Time using devices during telework	Pearson	−0.153	180*	−0.038	−0.019	−0.091
	Sig. 2-way	0.086	0.042	0.755	0.833	0.306
	N	127	127	127	98	127
Increased difficulty sleeping	Pearson	0.066	−0.331 **	−0.484 **	−0.045	−0.054
	Sig. 2-way	0.462	0.000	0.000	0.618	0.544
	N	127	127	127	98	127
Increased difficulty sleeping	Pearson	0.052	−0.209 *	−0.392 ***	−0.151	−0.117
	Sig. 2-way	0.614	0.039	0.000	0.137	0.251
	N	98	98	98	98	98
Devices used before sleep	Pearson	0.306 **	−0.007	−0.148	−0.168	0.007
	Sig. 2-way	0.000	0.940	0.096	0.058	0.938
	N	127	127	127	98	127
Age	Pearson	1	0.076	−0.128	0.048	0.182 **
	Sig. 2-way		0.396	0.153	0.594	0.041
	N	127	127	127	98	127
Sleep quality	Pearson	0.076	1	−0.210 *	−0.007	−0.027
	Sig. 2-way	0.396		0.018	0.938	0.760
	N	127	127	127	98	127
Time delay before sleep	Pearson	−0.128	−0.210 *	1	0.061	0.053
	Sig. 2-way	0.153	0.018		0.493	0.552
	N	127	127	127	98	127
Work organization	Pearson	0.048	−0.007	0.061	1	0.660 **
	Sig. 2-way	0.594	0.938	0.493		0.000
	N	127	127	127	98	127
Performance	Pearson	0.182 *	−0.027	−0.053	0.660 **	1
	Sig. 2-way	0.041	0.760	0.552	0.000	
	N	127	127	127	98	127

* Correlation is significant at the 0.05 level (2-way); ** Correlation is significant at the 0.01 level (2-way); *** Correlation is significant at the 0.001 level (2-way).

## Data Availability

Data related to this study’s materials are available in our archives of SPSS and respective syntaxes. Additionally, this work was not preregistered (not applicable). The project’s study was approved by the Ethics Committee of the Universidade Autónoma de Lisboa, Portugal.
